# Histopathological characterization of lung tumours at the University Teaching Hospital, Lusaka, Zambia: a pilot study

**DOI:** 10.4314/ahs.v22i4.5

**Published:** 2022-12

**Authors:** Mizinga Jacqueline Tembo, Violet Kayamba, Ephraim Zulu

**Affiliations:** 1 University of Zambia School of Health Sciences, Biomedical Sciences Department, Nationalist Road, P.O.Box 50110, Lusaka, Zambia; 2 Tropical Gastroenterology and Nutrition Group, University of Zambia School of Medicine, Department of Internal Medicine, Nationalist road, PO Box 50938, Lusaka, Zambia

**Keywords:** Lung cancer, histopathology, Zambia

## Abstract

**Background:**

There are limited data on histological classification of primary lung cancer from sub-Saharan Africa. Furthermore, the time trends of age-truncated incidence rates of lung cancer by histological phenotype in Zambia are also unknown.

**Objectives:**

The objective of this study was to determine histological types of lung tumours at the University Teaching Hospital (UTH) in Lusaka, Zambia.

**Methods:**

This was a retrospective pilot study of lung tumour biopsies collected from the histopathology laboratory at the UTH over a period of one year. Tissue sections were stained and when seen, lung cancer was classified using standard histological methods. Data were analysed using IBM SSPS version 23.

**Results:**

A total of 23 lung cancer tissues were retrieved. Histological types included eleven (47.8%) squamous cell carcinoma (SCC), six (26.1%) adenocarcinoma, two (8.7%) small cell carcinoma, two (8.7%) large cell carcinoma, 1 (4.3%) inflammatory myofibroblastic tumours and 1 (4.3%) pleural pulmonary blastoma. The results showed that the most affected age group was 60–69 years with most of the histological subtype in this age group being SCC. There was no statistically significant difference of histological subtypes across age groups, p=0.12.

**Conclusion:**

This study has shown that the most commonly diagnosed type of primary lung cancer is squamous cell carcinoma. More data are needed to further corroborate this observation.

## Introduction

Cancer of the lung is the leading cause of cancer related deaths globally. 1 In 2018, there were over 2 million reported lung cancer cases and about 1.8 million deaths. 2 There are currently very limited data on lung cancer from sub-Saharan Africa and the region is thought to have the lowest incidence rates globally. 2 Lung cancer is primarily classified into two major types; small cell lung carcinoma (SCLC) and non-small cell lung carcinoma (NSCLC) which can further be sub-classified into squamous cell carcinoma (SCC), large cell carcinoma (LCC) and adenocarcinoma (ADC). 3 Most SCLC and SCC arise from the central airways while ADC arises from the glandular cells of the peripheral airways. 4

On a global scale, SCC which was formerly the most common histologic subtype of NSCLC, has steadily fallen in incidence over the last few decades. 5 ADC has however, been seen to be the most prevalent histologic type of lung cancer so far, due to its rising incidence rates. 6 In Northern Africa, Lachgar et al., studied lung cancer cases between 2005 –2008 in Morocco and found that of all the lung cancer cases, ADC was the most prevalent type. 7 On the contrary, a study that was conducted in South Africa by showed that SCC was the most prevalent of their lung cancer cases. 8 The most common risk factor for lung cancer is cigarette smoking, but in addition, there are others including genetic factors, age, occupational hazards, radon exposure, radiation therapy and second-hand smoke. 9

Lung cancer evolves as a result of a series of mutational events and despite the studies that have been done in detail by numerous investigators, the molecular pathogenesis of lung cancer remains incompletely defined. 10 Many genetic alterations have been shown to be in related to lung cancer such as activation of epidermal growth factor receptor (EGFR), 11 mutation of Kirsten rat sarcoma viral oncogene homolog (KRA) or a tumour suppressor gene such as TP53 12, and the loss of the retinoblastoma protein (RB1) gene in the lungs. 13

In Zambia, the occurrence of histological sub-types of lung cancer has not been systematically evaluated. Estimated age standardised incident rates from the Zambia National Cancer Registry (a publication yet to be peer reviewed) rank it 21st with a case fatality rate of 33%. 14 However, GLOBOCAN ranks lung cancer as the 8^th^ most common cancer in Zambia. 2 There is an urgent need to conduct formal and systematic studies aimed at evaluation lung cancer in Zambia. In this study, we endeavoured to evaluate histopathological characteristics of lung cancers diagnosed over a period of one year at a fairly busy tertiary hospital.

## Methods

### Study Design

This was a laboratory based retrospective cross-sectional study at the University Teaching Hospital (UTH), department of Pathology and Microbiology, in Lusaka, Zambia. As standard of care, biopsy specimens taken from patients with suspected lung cancer are fixed in 10% neutral buffered formalin and embedded in paraffin wax in accordance with standard practice. These samples are evaluated by pathologists to determine the histological diagnosis and their reports recorded in electronic departmental files. The fixed samples in paraffin blocks are the stored in the archives.

For this study, we searched the electronic data base for cases of lung cancer and retrieved the corresponding paraffin blocks over one year. As the focus of the study was primary lung cancers, we excluded secondary tumours from the analysis. We also excluded samples that did not have patient details. We recorded demographic characteristics such (age and sex) of the patients from whom these samples were collected. Prior to sample processing, each archival lung tissue specimen was given a unique study identification number. Slide preparation was done using standard methods and stained with Haematoxylin and Eosin as previously described. 15 To confirm the histological diagnosis, a qualified histopathologist examined each slide and classified the cancers according to the World Health Organisation Classification system. We however, were unable to conduct Immunocyto/histochemistry on the samples as this was not a funded project.

Data were analysed using Stata version 15 (College Station, TX, USA). Proportions were summarised using frequencies and percentages. We used the Kruskal-Wallis test to evaluate any association between age groups and histological sub-types.

The University of Zambia Health Science Research Ethics Committee approved the study, ref: 20190217057.

## Results

### Demographic characteristics and histological type of lung tumours

We retrieved a total of 91 archival samples. Sixty-eight (75%) were excluded for being metastatic (secondary) tumours (n=63) or having missing patient details (n=5). The distribution of primary lung tumours was higher in males (70%). The median age for patients with primary lung cancer was 63 years (IQR 43- 71 years). Three of the patients were male children with ages 6, 8 and 12. The age group with the most disease burden of lung tumours was 60 – 69 years with a percentage of 30% and the age group with the least disease burden was between 30 – 39 years with a percentage of 4%. (Table 1) The most frequent histological type of lung tumour was found to be squamous cell carcinoma with a percentage of 48%.

**Table 1:** Table showing the demographic characteristics and histological type of patients whose samples were included in the analysis

Demographic characteristics	Number (Percentage)
**Sex** Males Females	16 (69.6) 7 (30.4)
**Age** Below 30 years 30–39 years 40–49 years 50–59 years 60–69 years Above 70 years	3 (13.0) 1 (4.3) 4 (17.4) 2 (8.7) 7 (30.4) 6 (26.1)
**Histological type** Squamous cell carcinoma Adenocarcinoma Small cell lung carcinoma Large cell carcinoma Pleural-pulmonary blastoma Inflammatory myofibroblastic tumour	11 (47.8) 6 (26.1) 2 (8.7) 2 (8.7) 1 (4.3) 1 (4.3)
**Total**	**23(100)**

### Histological classification by age group

The histological types among the children were myofibroblastic tumour in the 6-year-old, a blastoma in the 8-year-old and adenocarcinoma in the 12-year-old. Squamous cell carcinoma was highest in the 60–69 years age group (Table 2). There was no statistically significant difference in histological subtypes by age group (p=0.12).

**Table 2:** Analysis of lung cancer histological types by age group *Indicated in the tables are row percentages to show proportions within each age group

Age group	Blastoma n (%)	Myofibroblastic tumour n (%)	Small call Carcinoma n (%)	Squamous cell carcinoma n (%)	Adenocar cinoma n (%)	Large cell carcinoma n (%)
<30	1 (33%)	1 (33%)	-	-	1 (33%)	-
30–39	-	-	-	1 (100%)	-	-
40–49	-	-	1 (25%)	1 (25%)	-	2 (50%)
50–49	-	-	-	2 (100%)	-	-
60–69	-	-	1 (14%)	5 (72%)	1 (14%)	-
70–79	-	-	-	1 (33%)	2 (67%)	-
80–89	-	-	-	1 (33%)	2 (67%)	-
**Histological analysis by age group using Kruskal-Wallis**						*p*=0.12

## Discussion

This retrospective study of archival lung cancer tissue showed that the age groups with the most disease burden of lung tumours were those above 50 years. The small numbers of primary lung cancer tissues found suggest that this cancer is not common in Zambia.

Cigarette smoking is the single most important risk factor for lung cancer. The Zambia STEP report on non-communicable disease risk factors showed that 12.3% of Zambians were smokers, with most of them being males. 16 It is however clear that despite being a major risk factor, most of cigarette smokers do not develop lung cancer. 17 In addition, there is evidence that the lifetime risk for lung cancer among heavy smokers is less than 25%. 18 In their study of lung cancer in India, Noronha et al., concluded that a considerable number of Indians with lung cancer were not cigarette smokers. 18 Our intension is not to dispute the importance of cigarette smoke in lung cancer development, but merely to highlight the complexity and interplay of factors influencing carcinogenesis.

Our findings also showed a few lungs tumour cases in patients under the age of 30 years. However, we also found records of a 12-year-old with adenocarcinoma which is unusual at this age. One of the reasons why lung tumours were present in younger patients could have resulted from genetic mutations in the patients resulting in cancer. 19 Generally, lifestyle risk factors tend to cause cancer at more advanced ages. The sex distribution of lung tumour cases was reported to be higher in males than in females in this study. These findings were similar to studies done in Morocco, 7 Egypt 2 and Russia 2 where lung cancer cases were more common in males as compared to females. Males are more likely to be exposed to occupational carcinogens such as arsenic, 20 asbestos 21 and radon, 22 hence the possible reason for our findings. It could also have been as a result of certain lifestyle choices such as cigarette smoking and tobacco use, which are more commonly practiced by men.

The different histological types of lung tumours we found were squamous cell carcinoma, adenocarcinoma, small cell carcinoma, large cell carcinoma, pleural pulmonary blastoma and inflammatory myofibroblastic tumours. According to our study, squamous cell carcinoma was found to be the most common histological type of lung tumours and this was in agreement with the studies done in South Africa 8 and the United Kingdom 23. Squamous cell carcinoma is most commonly associated with smoking and tobacco use, despite information from the Tobacco Atlas database that states that Zambia generally has fewer smoking individuals as compared to other medium high development index countries. 24 These findings were different from those done in the United States by various authors who found adenocarcinoma to be their most common finding, 23,25 possibly because of changes in cigarette design that favour the development of adenocarcinoma hence giving a higher number of cases in the United States than in many other countries. 6

Amongst our findings, a case of the rare lung tumour type pleural pulmonary blastoma was seen. It mostly affects the younger population hence explaining why the patient was below 20 years of age, and this could have been due to familial germline mutations thus the development of this mesenchymal tumour. 26 We also found inflammatory myofibroblastic tumour which is also a rare tumour mostly found in children. 27 This tumour was the only benign tumour type that we found in our study.

In our study, we found that squamous cell carcinoma was commonest within the age range 60 to 69 years of age. This may have been attributable to the fact that this type is more common among elderly former or current smokers and tobacco users, and it may take many years in order for a cancer to manifest despite the presence of familial genetic mutations. 5 Inflammatory myofibroblastic tumours and pleuralpulmonary blastomas were found in those below 30 years of age and this could have been because they most commonly affect children. 26,27

We found that squamous cell carcinoma was more common in male patients whereas adenocarcinoma was more common in female patients. This was in agreement with a study done in China that showed similar findings. 28,29 Squamous cell carcinoma is more common in males because they make up the majority of the cigarette smokers while females, who make up the majority of the non-smokers, tend to have the adenocarcinoma type and this may possibly be a reason for our findings. 30

Different histological types, including rare types, of lung tumours were seen hence the findings proved to be clinically significant. The fact that we found different types of lung tumours signifies that our findings are clinically relevant because different types of lung tumours have different responses to treatment. However, there was no statistical significance found between the histological types of lung tumour with age or with sex, and this could have been due to the small sample size.

The major limitations of this study include the number of cases that we found. These small numbers could have could have led to inaccurate proportions of tumours studied and therefore, there will be need to conduct a more comprehensive longitudinal study of lung cancer in Zambia. Furthermore, specific cancer diagnostic methods such as immunohistochemistry would have provided additional and more precise characterization of lung cancer sub-types. We were not able to immunotype the tumours and did not therefore, evaluate for the expression of PD-L1 or provide information on specific mutation or ligand profiling.

## Conclusions

Different histological types of lung tumours were found and the most common histological type was squamous cell carcinoma. There was no statistical significance between the histological types of lung tumour and age groups.

## Figures and Tables

**Figure 1:**
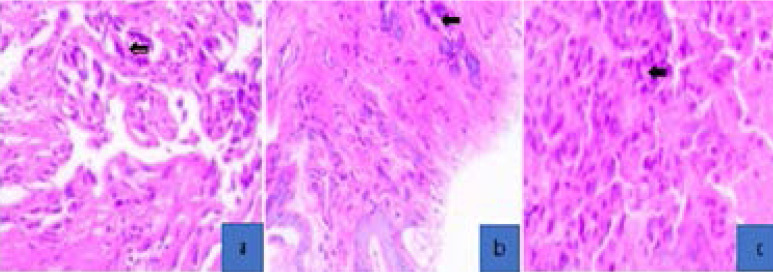
Photomicrographs showing various histological types of lung tumour (a) Adenocarcinoma, showing a glandular pattern of cancer cells; (b) Squamous cell carcinoma, showing an aggregation of squamous malignant cells; (c) Large cell carcinoma, showing characteristic large cells. Images at x40. (H & E staining)

## Data Availability

The histological characterisation and demographic data used to support the findings of this study are available from the corresponding author upon request.

## Supplementary materials

Nil.
